# Patients’ experiences from basic body awareness therapy in the treatment of binge eating disorder -movement toward health: a phenomenological study

**DOI:** 10.1186/s40337-019-0264-0

**Published:** 2019-10-21

**Authors:** Marit Nilsen Albertsen, Eli Natvik, Målfrid Råheim

**Affiliations:** 1 Department of Global Health and Primary Care, University of Bergen, Bergen, Norway; 2 Present address: Department of Eating Disorders, Division of Psychiatry, Haukeland University Hospital, Institute of Psychological Counselling , Bergen, Norway; 3 Department of Health and Caring Sciences, Western Norway University of Applied Sciences, Førde, Norway; 4 Department of Global Health and Primary Care, University of Bergen, Bergen, Norway

**Keywords:** Binge-eating disorder, Body-oriented therapy, Physiotherapy, Body experience, Phenomenology of the body

## Abstract

**Background:**

Binge Eating Disorder (BED) is the most common eating disorder. Patients with BED are often not diagnosed, nor offered adequate specific treatment. A great number of those who receive recommended treatment do not recover over time. More knowledge about central aspects of BED, and treatments that specifically target such aspects is needed. Previous research has linked body experience to the development and maintenance of eating disorders, as well as influencing treatment results and the risk of relapse. The aim of this study was to explore how patients with BED experience Basic Body Awareness Therapy (BBAT), which is a psychomotor physiotherapy treatment addressing body experience.

**Method:**

In this phenomenological study, we interviewed two patients with BED in depth during and after treatment. Video observations of treatment sessions and logs written by the patients were used as supporting data. The analysis was guided by Van Manen’s hermeneutic phenomenology.

**Results:**

A meaning structure was identified: “On the way from the body as a problem to the body as a possibility.” The two participants that besides BED also had a history of childhood trauma, perceived BBAT as a process of getting to know their own bodies in new ways, and described that the way they related to their own body changed as did aspects of their way of being. These changes were prominent when the participants described emotions, movement, pain, calmness, and self-experience, and interwoven with relational aspects as well as practices in everyday life.

**Conclusion:**

The present results indicate that BBAT stimulated body experience in a way that opened new possibilities for two participants with BED, and hence that BBAT can improve the health status of BED patients also suffering from childhood trauma.

## Plain English summary

Binge Eating Disorder is the most common eating disorder. Still, this disorder is often not addressed by the health care system, and current treatment shows poor results on a large group of these patients. Difficulties in relating to own body are linked to the development and maintenance of eating disorders in previous research, and seem to influence treatment results and the risk of relapse. Basic Body Awareness Therapy is a psychomotor physiotherapeutic treatment addressing the relation to one’s own body. In this study, we have explored in-depth the experiences of two patients with Binge Eating Disorder during their treatment-process with Basic Body Awareness Therapy. This study indicates that changes in how these patients related to their own bodies during the treatment processes were meaningful to them, and implied a movement toward well-being and accepting one’s own body. Findings from this study inspire more research on body awareness raising approaches in the treatment of patients with Binge Eating Disorder.

## Highlights

- BBAT may contribute to meaningful change toward better health and well-being for patients with BED.
- BBAT stimulates body awareness and acceptance through simple movements and grounding oneself in one’s own body.
- Being able to accept what feels unpleasant in the body may open new possibilities for the way in which patients with BED relate to themselves, others, and everyday life.

## Introduction

Binge-eating disorder (BED) is reportedly the most-common eating disorder (Supina, Herman, Frye, & Shillington, 2016). Health-related quality of life is significantly worse in BED patients than in both individuals without BED and a population with extreme obesity (defined as BMI ≥40 kg/m^2^) [3]. Patients with BED have more interaction with the health-care system than the general population, but research indicates that these patients are often not diagnosed nor offered adequate specific treatment [3, 17, 21, 50, 53, 61].

Psychotherapy is a recommended treatment for patients with eating disorders (Helsedirektoratet [The Norwegian Directorate of Health], [19]). The English guidelines recommend enhanced cognitive behavioural therapy (CBT-e) [12]. However, research shows that about 30% of patients with BED receiving the recommended treatment do not recover over time [12, 19, 29]. More knowledge about treatments that specifically target the central aspects of BED is needed.

Body image is a central aspect of eating disorders in general, and also in BED [8, 25]. Research has linked body-image disturbances to the development and maintenance of eating disorders. It has also been shown that such disturbances can influence treatment results and the risk of relapse to eating disorders generally [5, 6, 8, 13, 14, 18, 39, 40, 49, 51, 59]. Body image is a complex phenomenon that includes the evaluation and perception of one’s own body, but also thoughts about, feelings for, and attitudes toward one’s own body [8]. Body image is closely related to self-esteem, and body-image disturbances have long-term consequences on social, physical, and emotional development [60].

The need for more direct ways to address body image in treatment for eating disorders was emphasized in early studies [42]. Even though research in this area is scarce [8, 63], recent studies indicate promising results [16, 20, 24, 63]. Direct ways of addressing body image is still not systematized in therapy or in research. Physiotherapy is quite unusual to include in treatment of patients with eating disorders, even though physiotherapists have a wide array of approaches and techniques to address body image and body experience [38].

Basic Body Awareness Therapy (BBAT) is a type of psychomotor physiotherapy that falls between body-oriented treatment and psychotherapy. It is a physiotherapy approach within the movement awareness domain developed to bridge physical, mental, and relational health challenges [46]. BBAT is inspired by both Western and Eastern movement traditions [10, 11]. This body awareness training modality was first brought into physiotherapy by the Swedish physiotherapist Dr. Roxendal in the 1980s. It is further developed within the International Association of Teachers in Basic Body Awareness Therapy, and has since the 1990s been officially accepted and implemented in physiotherapy education in Scandinavia. BBAT has shown promising treatment results in psychiatry and mental health and is increasingly implemented in somatic health care such as rheumatology [32, 37]. The BBAT sessions create therapeutic learning situations for the patients that aim at exploring and integrating unity, flow and rhythm into daily-life movements such as lying, sitting, standing, walking and moving together [48]. Each exercise ends out by reflecting together with the therapist [32]. BBAT treatment is described in further detail in the method-section.

BBAT focuses on the whole person, enhancing body awareness and the quality of movements in attempting to promote health. Movement quality is seen as a unifying phenomenon and expression of health, representing a synthesis of biomechanical, physiological, psycho-socio-cultural, and existential aspects [48]. A systematic review of randomized controlled trials with different physical therapy interventions for patients with anorexia and bulimia nervosa found that the patients who received BBAT reported significant improvements in eating pathology and depressive symptoms [58]. There are many similarities between the main mechanisms in the different diagnoses of eating disorders [12], and body image disturbance is one of them [8, 25]. Therefore, gaining knowledge about body-oriented treatment approaches to BED is expected to be useful. To our knowledge no previous study has explored changes in body experience in patients with BED undergoing BBAT as part of their treatment. The purpose of this study is to explore how patients with BED experience BBAT, and in what ways and on what dimensions this body awareness therapy influenced them as described from their own perspective.

### Theoretical foundation

The phenomenology of the body was developed by the philosopher Maurice Merleau-Ponty. His central contribution is the idea that how we perceive our body is our mode of access to the world, and hence the primary mode for knowing the world [27]. Humans are embodied minds and minded bodies, and as bodily beings we also inhabit the world. Meaning develops in the relationship between humans and the social world, primarily through noncognitive, often unconscious, preverbal bodily sensations and experiences. Our intentionality, an eternally to-from relationship with our world, is a bodily intentionality based on inhabiting the world [4, 26, 27, 41, 56, 62]. Our bodily being is also characterized by a kind of ambiguity that is existential in character [27]. We live in our bodies unreflectively, acting as agents inhabiting our world while simultaneously experiencing our bodies within the world. We reflect upon our own body and being in the world by objectifying it in different ways.

When the body changes dramatically, as in illness, our own relationship with the body and world also changes. Illness can be experienced as a split between the body as subject and object that changes our relationships with the practical and social world and thereby our self-experience and usual way of being in the world. Illness often generates resistance. A kind of strangeness replaces the familiar, reducing our comfort and pleasure in being in our own body and world. This sense of being is related to our inevitable perishability, in contrast to life being a spontaneous and meaningful interaction when we are healthy [54, 55].

Phenomenology is a philosophical discipline. Empirical research approaches inspired by phenomenology include a phenomenological attitude of openness and wonder, and thorough reflection on the basic structures of human experience [57].

## Method

### Methodology, design and methods

This qualitative, longitudinal study explored some of the impacts of BBAT on body experience in two participants who met DSM-5 [1] criteria for BED. This choice of design was deemed suitable for exploring the phenomenon of body experience in depth while searching for context and connections [4, 33]. We followed the participants over time during multiple periods of data collection [34]. In-depth interviews were chosen since, from a phenomenological perspective, they provide a privileged access to a person’s experiences of his or her lifeworld [22, 57]. We also videoed one BBAT session with each participant. In addition, the participants wrote logs about the process, as is common in BBAT. In-depth interviews were the main data source, supported by video observations and patient logs.

### Participants

The participants were recruited using a purposive sampling method called criterion sampling [33]. The two included participants had been diagnosed with BED and admitted for treatment at a specialized unit for eating disorders based on certain criteria. The number of participants was based on sufficient information being obtained to answer the research questions [34]; in-depth explorations can involve only a few participants if they provide rich material and are analysed thoroughly [33].

Two female participants with BED were included in the study: Participant A was in her twenties and Participant B was in her forties. Both reported a history of childhood trauma related to attachment, bullying and one of them being sexually abused. They suffered physical and mental health issues in addition to severe obesity and BED. Participant A was working, but participant B had not been working after having children. Both lived together with their children and the children’s father. We have chosen Ann as pseudonym for participant A, and Claudia as pseudonym for participant B.

### Treatment

The participants took part in treatment at a specialized unit for eating disorders, where CBT-e is the usual treatment. BBAT is offered as part of the treatment for patients who may especially benefit from it, when physiotherapists are available. Both participants took part in individual BBAT sessions in addition to CBT-e weekly from the start of the treatment process. The treatment trajectories were adjusted according to their individual needs throughout the process.

CBT-e consisted of sessions with a psychotherapist where they used daily written real-time recordings to become aware of eating-disorder behaviour. The participants were gradually able to eat more regularly by writing plans for what and when to eat during the day, stop gaining and stabilizing their weight. In CBT-e they also addressed processes maintaining their eating disorder, such as over-evaluation of shape and weight, dietary restraint and sensitivity to outside events and moods [12].

BBAT sessions involve the physiotherapist enhancing contact with “self” by focusing on basic movement principles when performing simple everyday movements (lying, sitting, standing, walking, and relational movements), use of the voice, and a special kind of massage. Postural balance, free breathing, and mental awareness are seen as inseparable key elements for improving movement quality. In a therapeutic environment, the patient is invited to explore and integrate flow, elasticity, rhythm, and intentionality in coordinated movement in relation to time, space, and energy. The sessions include reflections around the present experience. The therapist assists the bridging between the experiences from the sessions and everyday life [28, 37, 47, 48, 52].

The sessions often started with the participant lying down straight out on her back on a mat. The therapist sitting beside her at level with the abdomen. The therapist invited her to become aware of the contact between body and ground, becoming aware of the body, part by part by simply noticing without searching for anything in particular, starting with the legs, moving upwards with open curiosity. Then the therapist invited her to try to experience the whole body as one unity. To become aware of the breathing, the hands (of the patient) were resting on the abdomen, following this little movement with the fingertips, freeing the breathing by trying not to direct it in any way, and doing some small stretching movements from the centre, along the midline of the body. Ending this sequence very slowly, giving the body time, stretching and yawning, and in her own time sitting up. Then, together with the therapist reflecting upon her experience. In the next sequence sitting on a simple stool, facing each other, they usually did something quite similar, contacting the body with awareness. With accepting curiosity, noticing how they related to the ground with feet and pelvic area, adjusting to the midline, freeing the breathing, closing up very slowly and reflecting together. The same body awareness raising elements were usually performed when standing, where they also included some qi gong movements. Qi gong is traditional Chinese exercises from martial art, characterized as “meditative movements”, that are widely practiced for their documented health benefits [2]. The BBAT-session often ended with a light massage, called BBAT-massage, with the participant in a lying down or sitting position.

### Data production

Our plan was to follow each participant for 40 weeks, since the treatment was stipulated to take 20 weeks, with a follow-up session 20 weeks later. Since treatment for both participants lasted for more than 20 weeks, due to their individual needs, we followed Ann for 55 weeks and Claudia for 47 weeks. Before treatment started, the first author conducted individual in-depth interviews with both participants. Ann was interviewed again at the end of treatment, and at follow-up. Claudia was interviewed again 47 weeks into treatment. Since she received bariatric surgery, the ongoing treatment with CBT-e and BBAT was paused, and the second (and last) interview with her took place 1 week before surgery. Flexible designs are usual in qualitative research, being continually adjusted to ensure that it remains consistent with the objectives of a study [34].

**Fig. 1:**
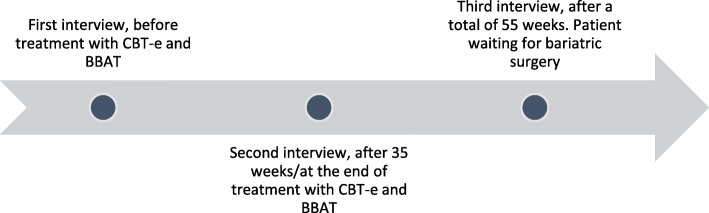
Timeline participant A (Ann)

**Fig. 2:**
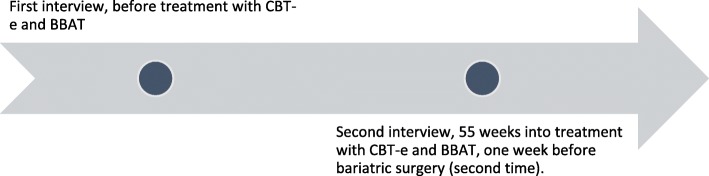
Timeline participant B (Claudia)

Each interview lasted about 1.5 h. A guide developed for each interview revolved around themes relevant to the research question: experiences from BBAT, changes, relationship with one’s own body, and possible relatedness between BBAT, body experience, and everyday life. With questions such as: “Can you tell me about your process with BBAT? Did you find it challenging in any way? Did you find aspects of the treatment especially useful or positive in any way?” Follow-up questions according to what the participants were telling, description of concrete situations to illustrate, as well as open questions were emphasized. The interviews were conducted in a treatment room of a specialized unit in calm and familiar surroundings. Each interview was audiotaped and transcribed verbatim. Figures 1 and 2 illustrate the timeline for each participant.

### Analysis

The data material consisted of the transcriptions from five in-depth interviews. The purpose of phenomenological analysis is to capture and describe essential meanings of the phenomenon under investigation. Meaning is always multidimensional, involving several layers. This analytic process aimed to illuminate structural and thematic aspects of the lived experience of patients with BED receiving BBAT.

Van Manen [57] distinguished between holistic and selective reading. Holistic reading involves reading the text as a whole while trying to capture an overall essential phenomenological meaning from the participants’ experiences, whereas selective reading involves reading the text several times, finding phrases or statements that seem particularly important for describing the phenomenon [57].

The first author wrote summaries after each interview, including descriptions of the mood and atmosphere. All of the authors read the interview material for each participant separately, using holistic reading, and then together discussed the core meanings. We then looked at the entire material from both participants, still using holistic reading, to determine an overall essential-meaning structure. A preliminary essential-meaning structure was identified. We then switched to selective reading, searching for phrases specific to the essential-meaning structure. Going back and forth between these two reading processes while reflecting and searching for meaning was the core part of the analysis.

The meaning structure that evolved and the five essential themes of the meaning structure are described in the Results.

### Reflexivity

The first author is a physiotherapist, a trained BBAT therapist, and has a long clinical experience of working with patients with eating disorders. The other authors are physiotherapists and senior researchers, but are not BBAT therapists and do not have any clinical experience with this patient group. Therefore, repeated discussion with the other authors together with other reflective practices were practiced to maintain an analytic distance and avoid drifting away from the lived experience of the participants.

## Results

The meaning structure “On the way from the body as problem to the body as possibility” is presented first. This is the overarching theme, a condensed description of the core content. This is followed by five sub-themes, “emotions”, “movement”, “pain”, “calmness”, and “self-experience”, that describe important nuances and variations in the participants’ treatment experiences and include quotes.

### On the way from the body as problem to the body as possibility

In the first interview, the participants described their body as a problem. They used words such as fat, heavy, ugly, and felt that the body took up too much space, was painful, made them restless, and was problematic when moving. It was difficult to identify themselves with a body like this, and it was an obstacle to them participating fully in daily life. They described the body as an object, something strange, like something being experienced from a distance. They were ashamed of their big body, and saw fixing the body through bariatric surgery as the only way out of the misery they were experiencing.

**Fig. 3:**
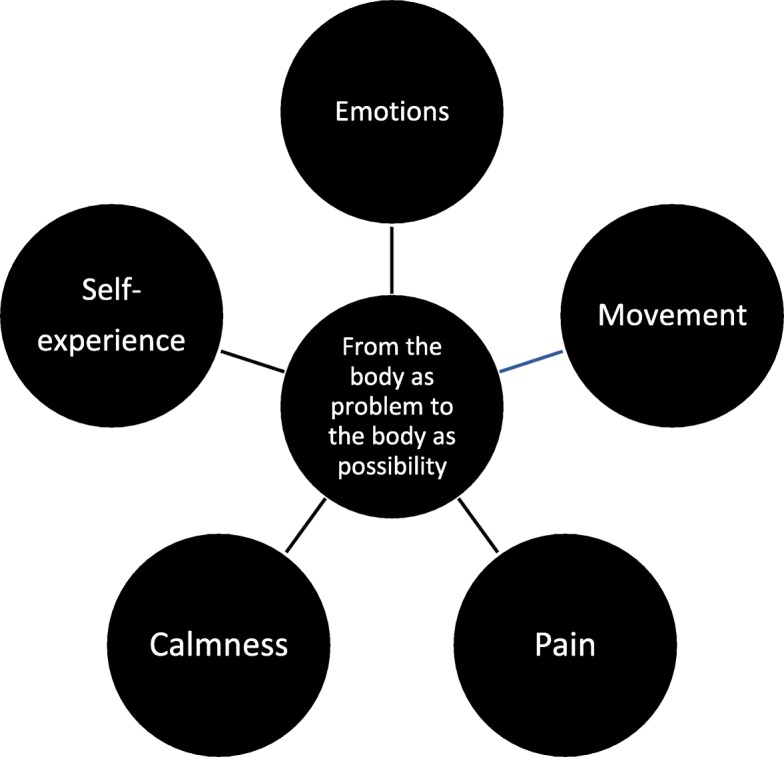
The meaning structure and its essential themes

The participants perceived BBAT as a process of getting to know their own bodies in new ways. When the participants were able to have new experiences in their own bodies, new possibilities were revealed. Changes occurred despite the body size, the wish to be thinner, the pain, the difficult emotions, and the cultural stigma related to obesity not going away. They went from hating their own bodies to being more accepting. First and foremost, this was related to a shift from trying to avoid attending to their own bodies and ignoring bodily sensations and reactions, to being more present in their own bodies while moving and acting. This development meant being able to take in and experience sensations and bodily reactions, and implied being less overwhelmed or controlled by fatness, emotions, or pain. New body-based experiences and recognitions became available to them since they were now more in contact with their own body. Moments of calmness and well-being stood out as significant experiences, and an evolving experience of their body and self as more unified opened new ways of relating to others and acting in their everyday lives. As illustrated in Fig. 3, the changes they experienced were especially prominent when the participants described emotions, movement, pain, calmness, self-experience in relation to others, and practices in everyday life.

### Emotions: from avoiding difficult emotions toward new emotional experiences

Both participants described that ever since they were small children they had used food to soothe themselves when experiencing uncomfortable emotions, which were often caused by difficulties in their lives. “I believe I have always done it. Like, when you are not fine, you can eat and then you will feel fine.” Their body became gradually larger, which resulted in hating their own bodies and made them feel ashamed and guilty. “I believe that I started to hate my own body when I was a little child.” These uncomfortable emotions were avoided through more binge eating, which became a kind of automatic response that they had little awareness of. They described themselves as caught in a vicious circle.

“My body is a result of how I have been through the years. So when I see my body, I think back on my past. Think of what made it this way. I walk around with a constant reminder of what happened.”

They also described their large body as a manifestation of how they had failed to take care of themselves, a proof of self-hate.

They saw bariatric surgery as the only solution to their problems, and it was in consultations at the hospital preparing for bariatric surgery that they both were diagnosed with BED. “This was the first time *eating disorder* was mentioned. It had never been talked about before.” Claudia had received bariatric surgery previously, but had regained the weight, and was preparing for a second operation. Ann was having her first operation. Even though it in one way felt like a relief to get the BED diagnosis, they were also afraid that the diagnose could become an empty excuse. Having to first receive conservative treatment with CBT-e and BBAT also led to disappointment and anger. “Afterwards I became a bit angry. Why has nobody brought this up ever in my entire life?”

The CBT-e for BED helped them to gain more control over the binge eating, by making plans for the day and eating regularly. At the end of treatment, they had stopped gaining weight and their weight had stabilized, which is a goal in CBT-e. The CBT-e and BBAT made them more aware of the relationships between food, the body, and emotions. Even though the size of the body stayed the same, the emotions related to their body and the capability to experience emotions in general had started to change. Ann expressed less hate toward her body, while Claudia became aware how much she hated herself because of her size; she now realized that even though she sometimes had imagined that she loved her own body, it was not true. This was a painful recognition, but also a precondition and a motivation for future change.

Then, I realized that what I say is just words. It is not the truth. … But I really want to, I hope, hope very much that I can change it. That I hope for. Because I am always a bit worried that I, my God I hate my body. I am a mother, a role model, and what I do will be reflected in my child. I don’t want her to adopt my mistakes.

Both participants described how this treatment process had taught them more constructive ways to cope with difficult emotions: “…But now I have found things I can do instead of comfort eating.” They could use elements from BBAT to calm down. Sometimes they started with listening to music, tidying the house, or talking to others before they could calm down through being present in the body here and now. This made it possible to endure emotional pain, and avoided turning to binge eating. However, sometimes they slipped back into old patterns with binge eating to avoid emotional pain. The ability of being present in their body facilitated more-positive emotions: “I feel a little in harmony, actually. I feel good, yes.”

During the treatment process, both participants seemed to acknowledge that it was necessary to treat the eating disorder before receiving surgery. Becoming slimmer would not necessarily solve their problems:

This was why I needed the operation so badly. To change it, to be able to be skinny. At the same time I thought “hallo,” the problems won’t go away, even though I become skinny. It is not in the head I will have an operation, it is actually in my stomach.

The participants did not believe that surgery and weight loss could solve all their difficulties. However, their current body size remained impossible for them to accept and live with, and both of them were still determined to receive surgery at the end of this process.

### Movement: from guilt and discomfort toward joy and well-being

Both participants had suffered from negative experiences in gym classes at school and health clubs. They felt physical activity was something they ought to do, it was painful, they did not cope, and often they ended up avoiding it. Physical activity was associated with duty and guilt. Their associations with movement were different. They described finding joy in everyday movements like walking, dancing, swimming, and playing with their children. Moving more and differently became more integrated with their everyday lives during the BBAT process, both at work and at home. Ann made small changes to her physical activity, such as choosing to walk up stairs rather than taking the elevator, changing position more often, and going for small walks. Movement could improve well-being: “It is lovely to be back after having a walk outside, and feel that I have done something good for my body. Oooh yes, it makes me feel more comfortable with myself.”

Ann also started to challenge herself in new ways, such as going hiking in the mountains because she felt a desire to do so. It was physically demanding, but she still got a lot of joy out of it:

I struggled and wanted to give up on the way up. It was completely horrible. Oh my God, what have I started, what am I doing? I found out that I am in terrible shape (laughing). My physical condition is non-existent. But, I look at it with a bit of humor. I struggled and struggled and struggled. …… And then, reaching the top…It was so delightful, damn (laughing)! It was so good! I think I have not been hiking in the mountains for at least 10 years. Since I finished secondary school. …It was so good, hah, I made it!

Performing new movements opened new possibilities in everyday life, from intense physical activity (as described above) to greater awareness of muscle tension, relaxation, and body posture. Claudia described how she used to activate her muscles by simply tensing up when sitting down with others, believing that this would prevent the chair from collapsing under her weight. Once she became aware of this, she learned how to relax her muscle tension. Ann discovered that she could sit down on the floor with her child. She had previously considered this impossible and so had never tried it due to the potential for feeling embarrassed if she would need assistance getting back up. However, now she could sit there for a while when adjusting her position, playing with her daughter, and get back up again on her own. She could attend to her child in new ways. This change was deeply valuable to her as a mother, she had previously felt shameful for passively observing her child play. To get down on the floor and play was important for their contact and interaction.

### Pain: from being controlled by pain toward living with pain through self-care

Both participants lived with pain, and worried that their body size could cause serious health problems. During the treatment process, they started to relate to pain in a different way that made them able to deal with it differently. Initially, they thought that there was nothing they could do when in pain other than taking painkillers. Now, they developed a different awareness of the pain, adjusting to it, trying to be more relaxed, and more able to cope with it. Claudia felt initially that the pain was her body telling her to take better care of herself. However, she ignored this even though she knew it was the wrong reaction. She found it difficult to take care of herself, which resulted in her pain intensifying. She slowly discovered that what she had learned in BBAT about adjusting her posture, letting go of muscle tension, breathing more freely, and being present and aware in her body reduced the pain: “I am actually constantly in pain. But, it is like when you do this (BBAT) it decreases. And, very often in these moments, it is actually really interesting, it feels as though the pain is letting go.” She found new ways to relate to the pain, where the pain did not absorb her awareness. The way she related to her own body, the way she moved, was a way to take care of herself.

Coping with pain became gradually more integrated in their everyday lives. One of the participants related this to the necessity of learning to take care of herself more generally: “The BBAT therapist made me realize that I need to function well with myself, in order to function well around others. So, I need to take care of myself. I have not been good at that.”

### Calmness: toward finding calmness in the body even in demanding situations

Both participants reported that they had a trustful relationship with the BBAT therapist. They found her calm, which positively influenced the sessions. They especially enjoyed the massage, and the exercises they learned helped them to relax in a new way that encouraged bodily well-being. They described this as more than just a change in muscle tone, using words such as delightful and wonderful: “Oooh it is so good. It is so delightful. …I get so relaxed.” They described the calmness stemming from BBAT as a new way of anchoring themselves in their body, which influenced how they experienced themselves and the surroundings.” My center, becoming aware of my breathing, being able to let go. A way of letting go of the outer world, while connecting to your inner world, if you might say it that way, actually my body.*”* The contact with the body and the awareness of inner resources was inter-related.

Both participants had busy lives full of responsibilities and stress. Finding calmness became a useful strategy in demanding situations. Ann started to go to the restroom at work when she just needed to be with herself. Claudia had always experienced problems when trying to put her child to sleep. She discovered that doing a “lying down exercise” from BBAT when putting her daughter to bed helped both her child and herself to calm down, enabling the daughter to fall asleep quickly. The participant described it almost like magic. She started to do this regularly, including in situations when she needed to calm her daughter down. Ann too described how she could calm down others by finding her own calmness: “I feel more in control. I am somehow trying to calm them both down (partner and child). And I feel I succeed better than I used to.”

Claudia described how she was able to take an MRI examination without taking valium for claustrophobia. She claimed that she would not have been able to do this without using what she had learned in BBAT about finding calmness in her body:

When having an MRI examination I would usually have said to my doctor that you have to give me something for my claustrophobia…But I managed to get through it by landing my whole body on the MRI machine, to find my centre, to find my breathing. I was actually so incredibly proud. Even though I hated it…I did not need to take a Valium pill, and I managed to do it on my own with the tools she (the physiotherapist) has given me.

One of the participants also used elements from BBAT to calm down when being overwhelmed with flashbacks from traumas. Avoiding discomfort through eating was a major problem for both participants. Hence being able to find calmness from within was of great significance. Learning how to calm down was described as one of the most positive aspects of BBAT.

### Self-experience: from self-hate toward finding strength from within

Initially the body size of the participants seemed to preoccupy their relationship with their own bodies. This influenced them when growing up, feeling limited by their body size in situations involving physical activity, not being able to dress as they wanted to, and avoiding certain social situations. During treatment, they seemed to open up to being more accepting of their own body. They were able to understand the body in new ways. One of the participants discovered that the hate toward her own body started after being sexually abused as a child. The other participant described how her body carried her history, and was a kind of reminder of difficulties from the past. One of the participants expressed how she started to relate to her body in a more accepting way: feeling safer and more familiar in her own body, and feeling more in balance. Both participants saw BBAT as a way of finding their true selves. They started to describe own body as part of their self, but still in a distanced language: “Him (my body) is not just there anymore. He has been included in me.” There was a movement towards integration, but still a distance between herself and her experienced body.

Claudia who had realized the hate toward her own body longed for feeling proud in, and of, her own body. As her perspective on her own body changed, she was less preoccupied by its size. “My body is just a part of me. The way I look is a temporary situation. But I have value from being who I am. And I mean something to myself and for the people around me.” In our last interview, she expressed that a more free and solid self-experience was related to letting go of the exclusively negative viewpoint of her body as being too big. Saying this out loud was an eureka moment for her. Gradually she felt more integrated with her own body, and this was associated with well-being and empowerment: “So I felt kind of ok, in here there is a lot like …Him (my power), just have to be released again, if I can say it that way.”

The participants felt like getting in touch with some kind of force from within. In these moments, the negativity toward their body size was less prominent. They felt more unified and stronger when opening up about their own body, and newly perceived their own body as a carrier of their life history, which increased their acceptance of their body:

Previously I often thought that I could have just changed my whole body. It would not matter. I would gladly have done it. I would have done it on the day. But now, I get like no, no. Because this body, it influences me. In a way it proves what I have been through. I do not want to get rid of my body now. Its mine. I know it sounds all weird, but…

This participant talked about resisting the usual goals to change one’s body, and that positive outcome usually is measured by this. Instead, she reclaims her body as part of the self – as having an energy of its own (“influences me”), holding and respecting her history as a survivor (“it proves what I have been through”) and a reclaiming of her body as her own (“it is mine”). Finally, her qualification at the end “I know it all sounds weird” suggests that conceptualizing her body on these terms is a different discourse to what she has previously been positioned by – a shift from changing my/her body towards reclaiming my/her body to be me.

Their increasing ease with their own bodies opened up new possibilities in everyday life. One of the participants described how she now dared to do things that she used to hate, like wearing a bikini when going to a spa hotel with her husband. It was difficult, but she could do it. She was able to realize that people have different shapes and most of them have complexes. She was working on stopping comparing herself to others and accepting the way she was: “Everybody has different shapes, and almost everybody also has complexes.”

Negative feelings toward her own body had previously prevented her from engaging socially. She started to put more effort into how she looked, and took up new activities and social engagements that she really enjoyed. In safe situations, it was easier to be themselves, especially with their children:

Usually I will say that my body and myself are not there in a way. But I believe maybe that when I am with my daughter, then it melts together. Then it is just me. And then it is all ok…

Being a mother was a large part of the identity of both participants, something they felt proud about. They really wanted to be good role models for their children. They wanted their children to feel good about their own bodies, and focused on transferring this during the treatment process. But, even at the end of this process, one of the participants expressed worries and guilt about what she was still unable to offer her daughter. Her body prevented her from participating in activities commonly associated with being a mother, such as going on rides in an amusement park, jumping together on a trampoline, or going downhill on sledges during wintertime. She was afraid that her child could be bullied because of the size of her mother. She longed for strong self-confidence, realizing that it would be of great value to her daughter.

The five essential sub-themes and the meaning structure are integrated, meaning that the sub-themes both describe the meaning structure in depth and are part of it. The participants expressed how the treatment initiated change in relation to own body, to self, and to surroundings and others. They experienced feeling better, even though they still felt the need to lose weight.

## Discussion

The findings of this study suggest that the participants experienced a change. They started out feeling fragmented, trying to avoid perceived unpleasant aspects about their body weight and shape, difficult emotions, own history, and pain, all rooted in the body, fighting themselves from within. Gradually they became able to accept these problems as part of who they had become, and started a process of feeling more integrated with their own bodies. We now discuss how this change was related to the treatment they received.

The participants described that in CBT-e they learned how to reduce the destructive eating patterns that they had previously used for coping with difficult emotions. Giving up habitual coping strategies, even destructive ones like binge eating, increases the need to receive training in new ways of coping, which in our case supports a synergistic combination of CBT-e and BBAT. In the BBAT sessions, the participants were encouraged to explore the sensations and reactions of their own bodies, and being present while performing simple movements with curiosity and acceptance. They reflected upon these experiences together with the therapist. Being aware of sensations, even when they are unpleasant, can be a new way of coping, as an alternative to avoidance. Instead of continuing to fight themselves from within, they started to get to know their bodies in new ways, by being more aware of aspects they previously had tried to avoid. As apparent in the participants’ narratives, becoming aware through acceptance of one’s own sensations may make a person able to get in touch with their own needs and resources.

From a phenomenological point of view, moving from avoidance to greater acceptance of one’s own body is a crucial shift, since the body is understood as our mode of access to the world [26, 27]. From this perspective it is understandable that negative feelings for and problems with one’s own body when performing practical tasks and socializing due to being overweight and experiencing pain and shame leads to experiencing a distance and alienation from the body and from certain dimensions of the everyday world. In this sense, discomfort and unpleasurable experience of being in their own body and world seemed to be the baseline condition in which the treatment process was started, which is how illness might be understood from a phenomenological point of view [55].

The female participants in this study had not suddenly fallen seriously ill, nor experienced a sudden marked bodily change. Instead, their habitual bodies were connected to different forms of objectifications and alienation that they had experienced. Objectification of one’s own body can indicate a split in experience between body and self [55]. The participants initially judged themselves as having fat bodies, and were very sensitive to others judging them as being too big. They described their present body shape as a temporary state, something they wanted to get rid of, and a preoccupation with their body size seemed to grow alongside their self-hate. However, they were still able to perform their usual activities such as taking care of their children and participating in working life. The experienced split was related only to some aspects of their own body and the everyday world. These kind of challenges from being obese, such as overwhelming physical sensations and body shame is also described in other qualitative studies [7, 43]. This current study adds to the knowledge base in describing bodily experiences as a potential for change, a therapeutic access, and highlighting the central role of the body in BED.

The change the participants described represented a movement from an experience of fragmentation to feeling more unified and in harmony with their body. According to a phenomenological understanding, these opposing experiences are both rooted in the ambiguity of our bodily existence [27]. The process of the change that emerged from the participant’s stories can also be understood as a movement from illness toward health.

BBAT is based on the clinical assumption that health issues may be related to a threefold lack of contact with the physical aspect of one’s own body, physiological, and mental processes, and with the environment which includes both the physical reality and relationships with others ([10], 1997). From a phenomenological point of view, the lived body is mutually interrelated with the lived space, time, and relationships. Splits of different kinds experienced between the body and self and the body and the world are anchored in this interrelationship [27]. In our context there seemed to be close links between experiencing integration between the body, self, and world, and increased contact with their own bodies. From a phenomenological perspective, the body has primacy in understanding and giving meaning to the world [26, 27]. Gendlin [15] emphasized how tuning in to one self or focusing inwards on one’s own body may lead to a deeper and wiser self. Gendlin described this “felt sense” in the body as the physical experience of meaning in life [15].

Integrating parts of self into a more cohesive sense of identity is the goal in a diverse range of psychotherapies [36]. As a slow integration of parts of self became apparent in the process of the participants, they describe a calmer and more anchored way of being. Being aware of present sensations, making contact with own breathing, rhythms, and touch, and tuning into oneself and others—as participants practice in BBAT—are also ways of regulating activation in the nervous system [23, 35, 44, 45]. Turning off the defence-systems and achieving a more-optimal physiological activation, referred to as the window of tolerance, makes one able to take in and be aware of the situation, to explore, reflect, and learn, and stimulates social engagement [30, 35, 44, 45]. The experiences of the two participants in this study appeared to reveal a link between being in contact with their own bodies, integration of self and more-optimal neurophysiological regulation. Self-integration, regulation, and the window of tolerance are well known concepts within the field of trauma, developmental psychology and attachment theories [30, 31]. Both participants had troubled childhood experiences, and the treatment processes with BBAT seemed to be useful in addressing their experiences of some of the adverse consequences of childhood trauma. BBAT encouraged a greater acceptance and a new familiarity with their bodies that these participants initially wanted to get rid of, and avoid. They did not actually start to like these aspects, but they were able to relate to them and expressed that they were more able to live with them. They started to relate more to their current situation, to the here and now. Their experiences resonate with findings from other eating disorder research where an improved familiarity with one’s own body was found to improve treatment outcomes [9].

## Conclusions

Our study points to CBT-e and BBAT representing a synergistic combination of two treatment approaches in the treatment of patients with BED. Addressing the patients` relationships with their own bodies in therapy—BBAT in this study— may improve their health status. These insights indicate that bodily approaches such as BBAT might be useful for patients struggling with BED and suffering from childhood trauma. The two participants` relation to own body was characterized by struggles with emotions, movement, pain, difficulties in relating to aspects of own history, body size, and shape.

Pragmatic validity refers to the relevance and usefulness of core findings and insights from qualitative studies in practice [22], for instance for clinicians treating these patients. Description of the essential dimensions of patients’ experiences from two treatment processes in this study, combined with insights from the discussion, might encourage clinicians to become more curious about, and open to explore bodily awareness raising treatment approaches such as BBAT in the treatment of patients with BED also suffering from childhood trauma. This study indicates that contact with own body seems to facilitate a more integrated self and improved health for these patients, and more research in this area is needed.

## Data Availability

To ensure full anonymity for the participants the transcribed interviews are not possible to share publicly.

## Abbreviations

BBAT: Basic Body Awareness Therapy
BED: Binge Eating Disorder
